# Developmental and Degenerative Characterization of Porcine Parthenogenetic Fetuses during Early Pregnancy

**DOI:** 10.3390/ani10040622

**Published:** 2020-04-04

**Authors:** In-Sul Hwang, Mi-Ryung Park, Hae-Sun Lee, Tae-Uk Kwak, Hwa-Young Son, Jong-Koo Kang, Jeong-Woong Lee, Kichoon Lee, Eung-Woo Park, Seongsoo Hwang

**Affiliations:** 1 Animal Biotechnology Division, National Institute of Animal Science, Rural Development Administration, Wanju, Jeonbuk 55365, Korea; insuri2642@korea.kr (I.-S.H.); mrpark45@korea.kr (M.-R.P.); leehs1498@korea.kr (H.-S.L.); ktu0206@korea.kr (T.-U.K.); pewkys@korea.kr (E.-W.P.); 2 College of Veterinary Medicine, Chungnam National University, Daejeon 34134, Korea; hyson@cnu.ac.kr; 3 College of Veterinary Medicine, Chungbuk National University, Cheongju, Chungbuk 28644, Korea; jkkang@cbu.ac.kr; 4 Biotherapeutics Translational Research Center, Korea Research Institute of Bioscience and Biotechnology, Daejeon 34141, Korea; jwlee@kribb.re.kr; 5 Department of Animal Sciences, The Ohio State University, Columbus, OH 43210, USA; lee.2626@osu.edu

**Keywords:** assisted parthenogenesis, pregnancy, fetal development, abortion

## Abstract

**Simple Summary:**

To increase the early implantation rate, oocytes and zygotes have been subjected to various artificial stimulations before and/or after in vitro fertilization, nuclear transfer, or sperm (spermatid) injection, etc. However, the stimulation process may induce parthenogenetic development. It is difficult to identify whether the embryo or fetus is normally fertilized or parthenogenetically activated in early pregnancy. In the present study, the porcine parthenotes originated from electric stimulation implanted and developed normally during the first month, in a manner similar to artificially inseminated embryos and fetuses. There were no statistical differences in the formation of the major organs such as the brain, liver, kidney, or heart in both groups. However, the implanted parthenotes radically ceased their development and degenerated after one month. It can be postulated that the parthenotes are one of the reasons for the gap between early pregnancy and delivery rate in assisted reproduction techniques.

**Abstract:**

The difference between early pregnancy and delivery rate is quite large in assisted reproduction techniques (ARTs), including animal cloning. However, it is not clear why the implanted fetuses aborted after the early pregnancy stage. In the present study, we tried to evaluate the developmental and morphological characteristics of porcine parthenogenetically activated (PA) embryos or fetuses by electric stimulation during the early pregnancy period. The implanted PA and artificially inseminated (AI) embryos and fetuses were collected at day 26 and 35 after embryo transfer, respectively. The developmental and morphological parameters in the PA embryos at day 26 were similar to the AI embryos. The size, weight, formation of major organs, and apoptotic cells were not statistically different in both embryos at day 26. However, the PA fetuses at day 35 showed ceased fetal development and degenerated with abnormal morphologies in their organs. The day 35 PA fetuses showed significantly higher apoptotic cells and lower methylation status in three differentially methylated regions of the H19 gene compared to their comparators. Therefore, the normal development of PA embryos and fetuses during early gestation could lead to these pregnancies being misinterpreted as normal and become one of the main reasons for the gap between early pregnancy and delivery rate.

## 1. Introduction

In assisted reproductive technology (ART) programs, physicians and scientists have used a variety of mechanical, electrical, and chemical stimuli that mimic the induction of cytoplasmic calcium oscillation needed to activate oocytes after intracytoplasmic sperm injection (ICSI) or round spermatid injection (ROSI), resulting in high fertilization and pregnancy rates [1,2].

Electrical stimulation is commonly used to activate mammalian oocytes, along with other methods, including other ART methods [3,4,5] and cell fusion for somatic cell nuclear transfer (SCNT) in mammals [6,7]. Electrical stimulation triggers calcium influx by forming pores in the plasma membrane to initiate a second meiotic cell cycle [8,9]. It can also activate oocytes for the generation of parthenogenetically activated (PA) embryos.

Although the influence of paternal factors derived from sperm during fertilization is very important in embryonic and placental development, parthenogenetic embryos and fetuses can be generated to serve as robust models to study embryonic development and embryonic-maternal recognition during pre- and post-implantation stages. In addition, some of the paternally expressed genes that are abundantly expressed in the placenta contribute to normal development of the placenta [10]. However, parthenogenetic embryos without paternally expressed imprinted genes can be implanted and developed to the fetus stage up to early pregnancy [11]. In pigs, PA embryos have been co-transferred with SCNT embryos to increase the pregnancy rates of the SCNT embryos by increasing fetal-maternal recognition and communication from an increased number of total implanted embryos [6]. In a previous study, pig PA embryos derived from oocytes obtained by in vitro maturation (IVM) and activated by electrical stimulation could still be detected by ultrasonography after one month of gestation [12].

The percentage of cycles resulting in pregnancies (21.2%, 6.3% to 35.9%) is usually higher than the percentage of cycles resulting in live births (16.34%, 3.2% to 34%) with ART in humans [13]. This gap may reflect miscarriages or stillbirths during pregnancy, especially during the first trimester. First-trimester miscarriages are more often due to abnormal embryonic development rather than maternal inability to maintain a normal pregnancy [14].

We hypothesized that one of the reasons for the gap could be PA embryos induced by artificial stimulation during ART procedures. In the present study, we tried to evaluate the developmental and morphological characteristics of porcine PA embryos and fetuses by electric stimulation during early pregnancy.

## 2. Materials and Methods

### 2.1. General Information and Ethics Statement

All chemicals used in the present study were purchased from Sigma-Aldrich Chemicals (St. Louis, MO, USA) unless otherwise stated. Additionally, in the present study, the protocol and standard operating procedures for the treatment of the pigs were reviewed and approved by the Institutional Animal Care and Use Committee of the National Institute of Animal Science, RDA (approval no. NIAS20181302).

### 2.2. In Vitro Maturation

The IVM protocol was performed as follows [11,15]. Briefly, ovaries were obtained from prepubertal gilts at a local slaughterhouse (Nonghyup Moguchon, Gimje, Korea) and transported to the laboratory within 1 h in saline at approximately 30 to 35 °C. Cumulus-oocyte complexes (COCs) were aspirated from follicles (3–6 mm in diameter) using an 18-gauge needle attached to a 10-mL disposable syringe. Follicular fluid with COCs was collected into conical tubes and washed three times in tissue culture medium (TCM)-199 (Thermo Fisher Scientific, Waltham, MA, USA) containing 0.1% (*w/v*) polyvinyl alcohol. After sedimentation, COCs consisting of several layers of compact cumulus cells were selected for IVM. Then, approximately 50–70 COCs were transferred into 500 μL of TCM-199 supplemented with 10% porcine follicular fluid, 3.05 mM D-glucose, 0.57 mM cysteine, 0.91 mM sodium pyruvate, 0.5 μg/mL FSH, 0.5 μg/mL LH, 10 ng/mL EGF, 75 μg/mL penicillin G, and 50 μg/mL streptomycin in a four-well dish. The COCs were cultured for 22 h with hormones and then 22 h without hormones at 39 °C under 5% CO_2_ in air. After IVM, cumulus cells were removed from oocytes by treatment with 0.1% hyaluronidase for 5 min. An extrusion of the first polar body was checked comprehensively, and oocytes with the first polar body were defined as mature and ready for use in further experiments.

### 2.3. Generation of PA and AI Embryos

Matured oocytes were activated electrically in medium containing 0.3 M mannitol, 1.0 mM CaCl_2_, 0.1 mM MgCl_2_, and 0.5 mM HEPES. In between electrodes 0.2 mm in diameter, two direct current pulses of 1.25 kV/cm were applied for 30 μs at 1 s intervals using an Electro Cell Fusion generator (Nepa Gene, Ichigawa, Chiba, Japan). The PA embryos were washed twice and cultured in porcine zygote medium-3 in a four-well dish at 38.5 °C under 5% CO_2_ in air. At least 2 h after electrical activation, the PA embryos were transferred into both oviducts of recipient pigs (Landrace x Yorkshire) on the same day or 1 day after the onset of estrus. As a control, artificial insemination (AI) was performed using commercial liquid semen purchased from a local artificial insemination center (Korea Pig Genetics, Gimje, Korea). The day of embryo transfer and artificial insemination was counted as day 0. Then, 26 and 35 days after embryo transfer and artificial insemination, embryos and fetuses were recovered from the uterine horn of recipients, respectively. The size and weight of all embryos and fetuses were measured and recorded.

### 2.4. Karyotype and Histological Analysis

Both the PA and AI embryos and fetuses collected were washed three times in PBS (Thermo Fisher Scientific, Waltham, MA, USA) containing antibiotic solution (1%, *v/v*) and cut in PBS containing 0.5% trypsin-EDTA solution (Thermo Fisher Scientific, Waltham, MA, USA). Then, the fetuses were enzymatically digested with 0.5% trypsin-EDTA in PBS for 30 min at 37 °C. The digested tissues were washed, and the cells released were cultured to confluence. Finally, a karyotype analysis was performed at GenDix (http://www.gendix.com, Seoul, Korea) after several passages using a standard high-resolution G-banding method. In preparation for the histological analysis, both PA and AI embryos and fetuses were fixed in 10% buffered formalin, embedded in paraffin, and sectioned into 4-µm slices, and then sections were stained with hematoxylin and eosin (H&E). Two pathologists analyzed the histological and pathological examination at Biotoxtech (http://www.biotoxtech.com, Cheongju, Korea).

### 2.5. Analysis of Apoptosis in Fetuses

Paraffin-embedded embryos and fetus slices on slides were deparaffinized by more than two treatments with xylene for 5 min each and then dehydrated by serial dilution in ethanol and distilled water. The slides were washed twice in PBS supplemented with 0.1% polyvinylpyrrolidone and permeabilized with 0.5% of Triton X-100 for 30 min at room temperature. A TUNEL assay was applied to assess the presence of apoptotic cells using an In Situ Cell Death Detection Kit in accordance with the manufacturer’s instructions. After the TUNEL reaction, slides were covered in ProLong Antifade Mountant with DAPI (Thermo Fisher Scientific, Waltham, MA, USA). The slides were maintained at −20 °C, and the numbers of apoptotic cells were determined by counts from randomly selected images obtained under an epifluorescence microscope (Nikon, Tokyo, Japan).

### 2.6. Methylation Status of Fetuses

A bisulfite modification and sequencing analysis to compare methylation profiles between PA and AI fetuses was conducted according to methods outlined previously [16]. Briefly, all four genomic loci were selected for amplification: one differentially methylated region (DMR) in the IGF2 gene and three DMRs (DMR1, DMR2, and DMR3) in the H19 gene (Figure S1). The bisulfite-treated DNA samples were amplified in two rounds of PCR with fully nested primers for each DMR region. The primer sets were specific for bisulfite-treated DNA (Table S1). PCR with Diastar EF-Taq DNA polymerase (Solgent, Daejeon, Korea) was performed as follows: 2 min at 95 °C, followed by 37 cycles of 20 s at 95 °C, 40 s at 55 °C, 30 s at 72 °C, and a final 5 min at 72 °C. The PCR products were extracted using a gel purification kit (Bioneer, Daejeon, Korea). Finally, the PCR products were cloned into T-vectors (Promega, Madison, WI, USA), and individual clones were sequenced by Genotech (http://www.genotech.co.kr, Daejeon, Korea).

### 2.7. Statistical Analysis

Each experiment was repeated at least three times. Data were analyzed by ANOVA followed by Fisher’s protected least significant difference (StatView 5.0 software, SAS Institute Inc., Cary, NC, USA). Differences were accepted as significant when *p* < 0.05.

## 3. Results

### 3.1. Developmental Characteristics of PA Embryos and Fetuses

**Figure 1:**
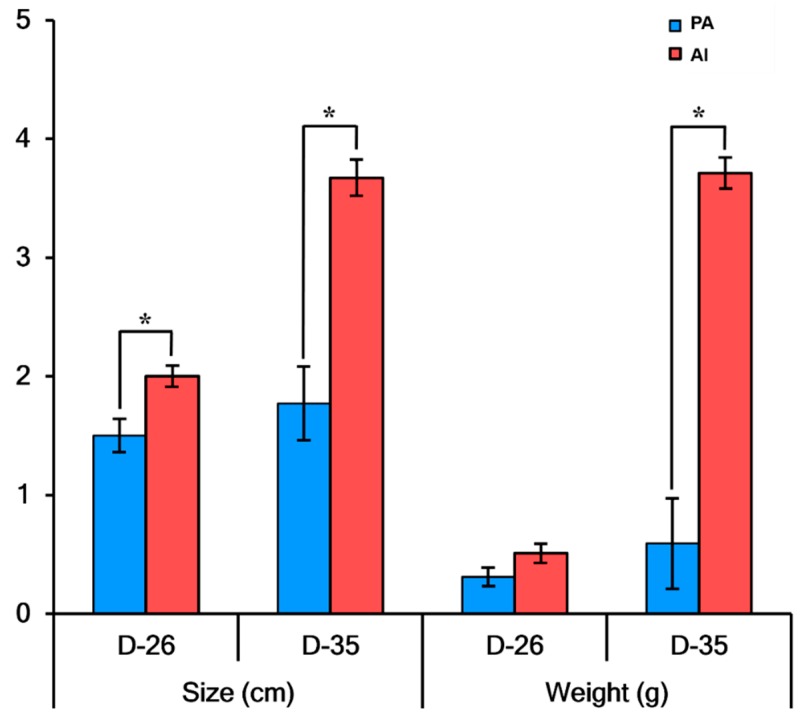
Sizes and weights of parthenogenetically activated (PA) and artificially inseminated (AI) embryos and fetuses. PA and AI embryos and fetuses were collected at day 26 and day 35 after embryo transfer. Immediately after collection, the embryos and fetuses were washed and their developmental characteristics such as size and weight were examined. * PA vs. AI (*p* < 0.05).

As shown in Figure 1, the weights of PA embryos (*n* = 15) at day 26 were not statistically different from those of AI embryos (*n* = 18), but PA embryos were significantly smaller than AI embryos (*p* < 0.05). The weights and sizes of PA fetuses (*n* = 9) at day 35 were statistically different from their AI counterparts (*n* = 3) (*p* < 0.05).

### 3.2. Histological Characteristics of PA Embryos and Fetuses

**Figure 2:**
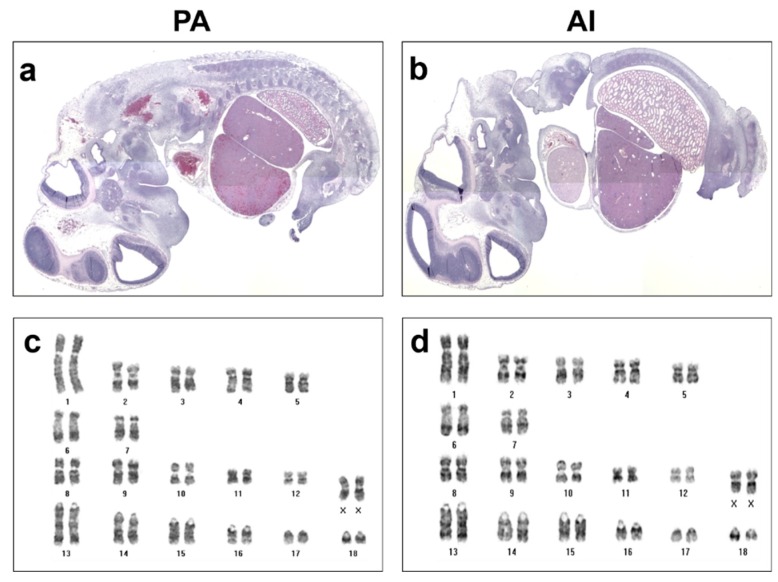
Developmental characteristics of PA and AI embryos. PA and AI embryos at day 26 were embedded in paraffin and sectioned in sagittal planes (**a**,**b**). Cell lines were established from both PA and AI embryos and subjected to karyotyping (**c**,**d**).

In Figure 2, histological examination of PA (a) embryos at day 26 showed normally formed major organs such as brain vesicles, hearts, livers, and kidneys compared to those of AI embryos (b). Interestingly, PA (c) embryos showed normal karyotypes, with 18 pairs of homologous chromosomes and 1 pair of sex chromosomes, which were the same as those of female AI (d) embryos.

**Figure 3:**
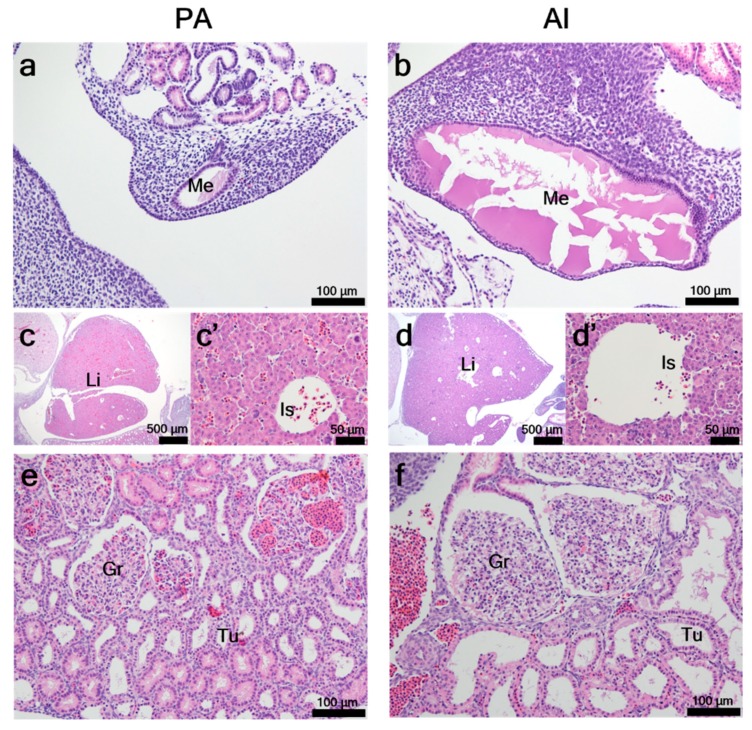
Histopathological analysis of PA and AI embryos at day 26. Histopathological examinations of developing mesonephric ducts (**a**,**b**), livers (**c**,**d**), and kidneys (**e**,**f**) were conducted on PA and AI embryos at day 26 after embryo transfer. Me: mesonephric duct; Li: liver; Is: liver island; Gr: glomerulus; Tu: mesonephric tubules.

PA embryos at day 26 showed the formation of mesonephric ducts in distal parts of primitive kidneys. The mesonephric ducts of PA embryos were in the formation rather than mature stage (Figure 3a), whereas in AI embryos they were in an expansion stage before the formation of metanephroses (Figure 3b). Under low magnification, the livers of PA embryos (Figure 3c) appeared morphologically immature when compared to those of AI embryos (Figure 3d). Under high magnification (Figure 3c’,d’), liver islands formed from primitive liver cells, but sinusoids were not yet fully developed in either PA or AI embryos. Kidneys in both PA and AI embryos showed normal development of glomeruli and mesonephric tubules. However, the expansion rate of mesonephric tubules and growth rate of glomeruli seemed lower in PA embryos (Figure 3e) than in their counterparts (Figure 3f).

**Figure 4:**
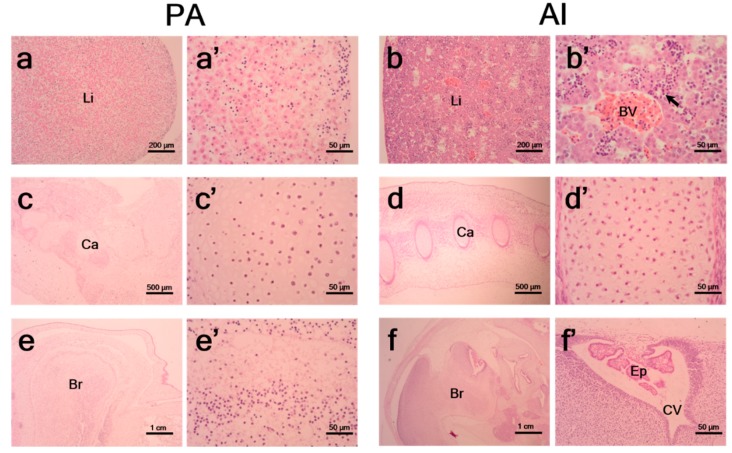
Histopathological analysis of PA and AI fetuses at day 35. Histopathological examination of developing livers (**a**,**b**), cartilage (**c**,**d**), and brains (**e**,**f**) were conducted on PA and AI fetuses at day 35 after embryo transfer. Black arrow indicates primitive hematopoietic cells in a liver section (**b’**). BV: blood vessel; Ca: cartilage; Br: brain; Ep: ependymal cell; CV: cerebral ventricle.

PA fetuses at day 35 showed clear morphological degeneration of livers (Figure 4a), cartilage (Figure 4c), and brains (Figure 4e), whereas AI did not (Figure 4b,d,f, respectively). The PA fetuses showed no signs of blood vessels in livers (Figure 4a’) and lower densities of cells in cartilage (Figure 4c’) compared to those in AI fetuses (Figure 4b’,d’), respectively. Unlike AI fetuses, PA fetuses showed degenerative cerebral ventricle and ependymal cells (Figure 4e’,f’). 

### 3.3. Analysis of Apoptosis in PA Embryos and Fetuses

**Figure 5:**
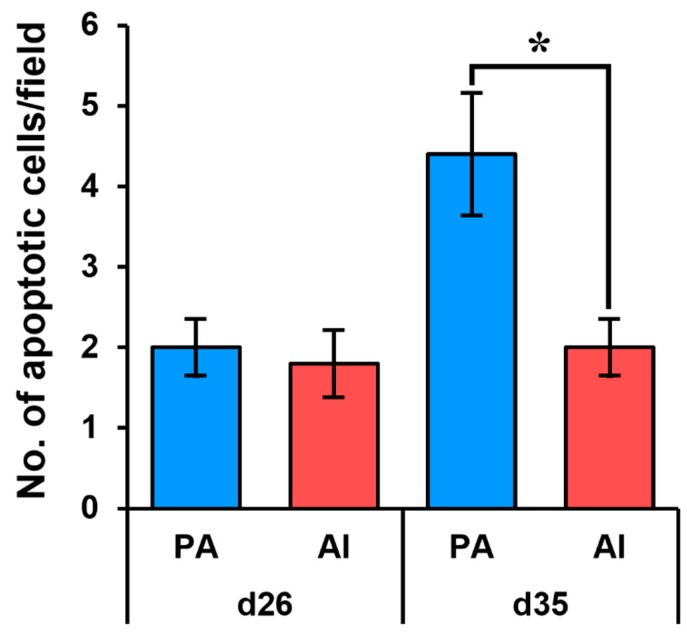
Analysis of apoptosis in PA and AI embryos and fetuses. An in situ cell death detection assay (TUNEL) was performed to measure apoptotic cells in PA and AI embryos and fetuses. The number of apoptotic cells was determined based on observation of a random field. * PA vs. AI (*p* < 0.05).

Based on the TUNEL assay, the rates of apoptotic cells in PA embryos were similar to those in AI embryos at day 26. However, the rates were significantly increased in PA fetuses at day 35 compared to those of their counterparts (Figure 5).

### 3.4. DNA Methylation Patterns in PA Fetuses

**Figure 6:**
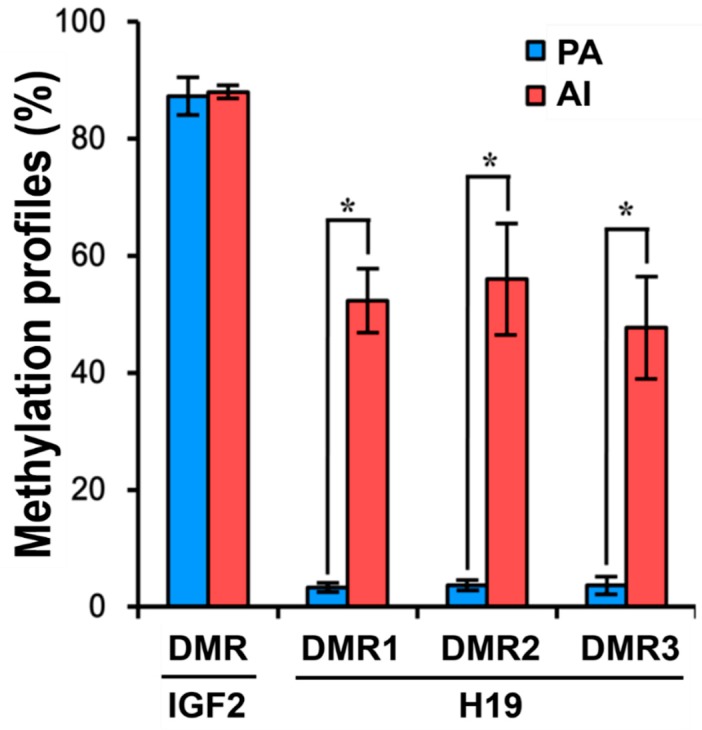
CpG methylation profiles of differentially methylated regions (DMRs) in IGF2 and H19 genes. The relative positions of DMRs in IGF2 and H19 are shown in Supplementary Figure S1. Results of a statistical analysis of the differences in methylated CpG sites in the IGF2 DMR and H19 DMRs are shown for PA (*n* = 3) and AI (*n* = 3) fetuses. The data are shown as means ± SEs of three replicates. * PA vs. AI (*p* < 0.05).

The methylation profiles of IGF2 showed no differences between PA and AI fetuses (87.3% vs. 88.0%). However, the methylation profiles of H19 showed significant differences between PA and AI fetuses in DMR1 (3.3% vs. 52.3%), DMR2 (3.7% vs. 56.0%), and DMR3 (3.7% vs. 47.7%) (*p* < 0.05) (Figure 6).

## 4. Discussion

The mean gap between the rates of pregnancies and live births is approximately 5% with ART treatment in humans [13]. Although this gap is partly attributable to abnormal embryonic development [14], little is known about parthenogenesis derived from artificially activated oocytes. Parthenogenesis is distinct from pseudo-pregnancy, and we hypothesized that PA embryos have contributed to the pregnancy–live birth gap with ART. In our previous reports, the early pregnancy of transgenic cloned pigs was 32% in alpha 1,3-galactosyltrasnferase knock-out with membrane cofactor protein knock-in and 44% in human CD73 transgenic, but the delivery rate was 16% and 17%, respectively [17,18].

To test this hypothesis, we produced PA embryos by electric stimulation, collected the embryos and fetuses from recipient pigs at day 26 and 35 after embryo transfer, and analyzed the developmental and degenerative characteristics of these embryos during early-stage pregnancy in pigs. The recipients classified as pregnant by ultrasonography had formed several normal gestational sacs. Day 26 PA embryos showed normal morphologies and histological similarities with AI embryos; after this, however, the PA embryos quickly degenerated. A previous report revealed that pig PA fetuses derived from in vitro matured oocytes activated by an optimized electrical protocol could be detected until approximately day 50 of gestation [12]. Based on Carnegie stages [19], day 32.5 of pig fetus gestation is comparable to day 58 of human fetal development [20,21], with day 50 of pig fetus gestation considered as comparable to the end of the first trimester in humans.

A minimum of four to five viable embryos [22] or at least 50% uterine occupancy [23] is required for assisted pregnancies to be maintained in pigs. However, the developmental capacity of SCNT embryos is not comparable to that of normal embryos [24]. To overcome this developmental shortcoming, SCNT embryos were co-transferred with PA embryos induced by artificial activation to increase the possibility of pregnancy [6,25]. The use of this uncommon treatment only in pigs is based on the normal development and degradation of PA embryos at an early stage in pregnancy. Normal development of the transferred PA embryos might help maintain uterine conditions for implantation of SCNT embryos. After natural degeneration of the implanted PA embryos, transferred SCNT embryos remain until delivery. As in previous studies, we used PA embryos to generate transgenic cloned piglets [19,26].

In the present study, PA embryos developed with nearly normal morphologies and implanted successfully by day 26 after embryo transfer, although PA embryos were smaller than the controls. These results were consistent with those of previous studies in pigs [17] and dogs [27]. It is difficult to find any abnormalities or deformities in fetuses in gestational sacs by hormonal examination and ultrasonography. They can be only estimated by the formation and maintenance of the sacs in uteruses. In addition, plasma estrogen levels were not different between the two groups (data not shown).

Based on the histological analysis, there were no differences in the formation of major organs such as hearts, livers, kidneys, and brains at day 26 in PA and AI embryos. Parthenotes of other species have also shown normal embryonic and/or fetal development to day 10 in mice [28], day 21 in sheep [26], and day 30 in dogs [27]. We also evaluated the development of primitive reproductive tracts in PA embryos. Although mesonephric tubules formed in both embryos, no or less glomerulus formation was observed in the tubules of PA embryos than in those of AI embryos. We could not confirm the formation of gonads in PA embryos because of limited embryonic samples. However, reproductive development in PA embryos appears to be similar or delayed relative to that in AI fetuses, but they may not be fully functional. To our knowledge, this is the first report on the formation of mesonephroses in PA embryos.

Nevertheless, at day 35 of development, PA fetuses showed detrimental morphological degeneration with no signs of blood vessels in livers, low cell densities in cartilage, and defection of cerebral ventricles and ependymal cells in brains compared to those in their comparators. Although it is difficult to determine the exact timing of the developmental degeneration of PA fetuses, it can be inferred that degradation has begun during fetal development due to the impaired imprinting status of these fetuses. Although many researchers have tried to investigate differences between PA and AI embryos, information on the reason for and time at which PA embryos cease to develop is still limited. In contrast to day 26 PA embryos, the total RNA extracted from PA fetuses at day 35 did not pass the quality control test. (Hwang et al. Animal Biotechnology Division, National Institute of Animal Science. Genomic DNA extraction from fetuses. Unpublished data, 2018).

Apoptosis (i.e., programmed cell death) plays a pivotal role in maintaining cell homeostasis in normal pre-implantation embryos and in mammalian reproduction overall. Its function includes the elimination of cells that develop abnormally to maintain a suitable number of embryonic cells [29] and regulation of cell growth and death in embryogenesis, organ metamorphosis, and tissue homeostasis [30,31]. This incongruity in the regulation of both cell growth and death during embryogenesis could be a reason for developmental failure in PA fetal pigs, although they appear morphologically normal. In the present study, day 35 PA fetuses exhibited a significantly higher number of apoptotic cells than their counterparts. Unlike at day 35, PA embryos at day 26 showed no difference in the proportion of apoptotic cells from those in AI fetuses. The regulation of apoptosis in PA fetuses, therefore, may not be initiated or may only be weakly initiated. The exact time point when the regulation of apoptosis begins in PA fetuses is not known.

Parthenotes cannot develop to full term because they develop without paternal chromosomes, resulting in abnormal imprinting [32,33] and failed placental growth [34]. The only case of full-term development of parthenogenetic offspring has been reported in mice with a 13-kilobase deletion in the H19 gene [35]. Aberrant expression patterns of imprinted genes are considered a primary reason for the developmental failure of PA fetuses [36,37]. The controlled expression of imprinting genes such as IGF2 and H19 plays a major role in embryonic and placental development in mammals. IGF2/H19 loci in pigs contain three DMRs with one CCCTC-binding factor (CTCF) binding motifs in the promoter region of H19 and two in DMRs of IGF2 [38].

According to our bisulfite-sequencing PCR results, H19 DMRs 1/2/3 were significantly hypomethylated and the IGF2 DMR was hypermethylated in PA fetuses compared to their counterparts. Our results are quite similar to those of a study on SCNT fetuses that originated from parthenogenetic somatic cells [39]. The SCNT fetuses also showed low methylation in H19 DMRs and high methylation in IGF2 DMR, compared to those in normally fertilized controls.

It is well known that the paternally expressed genes are important in placenta development, resulting in successful embryonic and fetal development [10]. In addition, the paternal factor alone does not have the ability to support embryonic development to full term, in accordance with a recent study using androgenetic embryos [40]. These findings suggest the importance of maternal and paternal factors in normal fetal development. In agreement with this, in the present study, the parthenogenetic embryos could be implanted and developed until day 23 of the Carnegie stage without the paternal factor, but degraded thereafter.

## 5. Conclusions

Taken together, these data indicate that the developmental and degenerative characteristics of PA embryos and fetuses were comparable to those of AI embryos and fetuses in early-stage pregnancy. The embryos showed similar gestational sacs, weights, and formation of major organs, as well as primitive reproductive tracts at day 26 after embryo transfer. However, by day 35, PA fetuses had degenerated, with high rates of apoptosis and low levels of methylation in developmentally relevant genes. Therefore, the normal development of PA embryos and fetuses during early gestation could lead to these pregnancies being misinterpreted as normal and become one of main reasons for the gap between early pregnancy and delivery rate.

## Supplementary Materials

The following are available online at https://www.mdpi.com/2076-2615/10/4/622/s1. Supplementary Table S1: PCR primer sets for bisulfite sequencing, Figure S1: Relative positions of the Igf2 and H19 DMRs.
