# Supplementary material for: Developmental and Degenerative Characterization of Porcine Parthenogenetic Fetuses during Early Pregnancy
Source: Animals (Basel). 2020 Apr 4;10(4):622. doi: 10.3390/ani10040622 (PMC7222715; doi:10.3390/ani10040622)
Supplement: Supplementary file 1 [file animals-10-00622-s001.zip › animals-742082 -supplementary/Supplementary Figure 1-edited.docx]

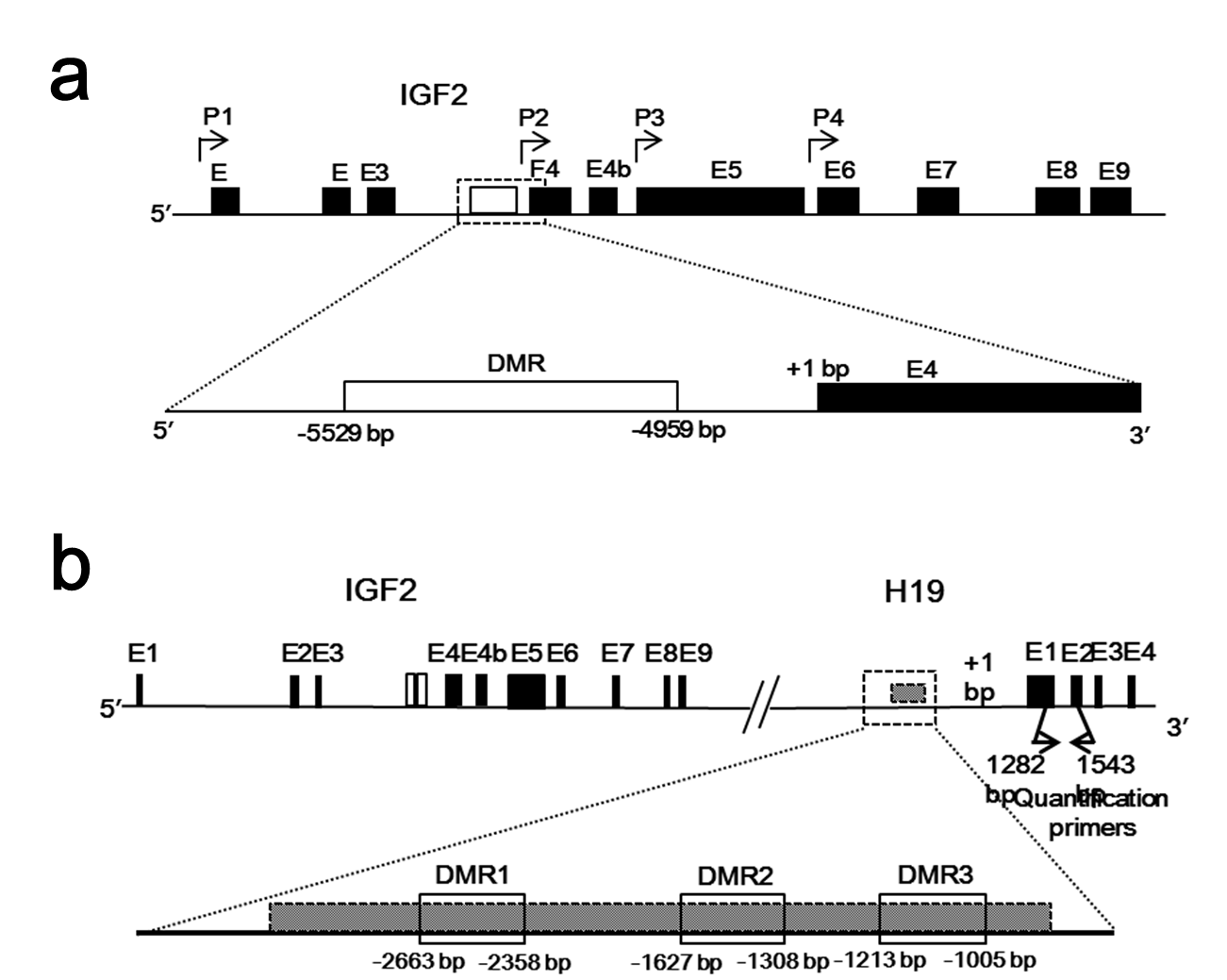


**Supplementary Figure 1.** Relative positions of the *Igf2* DMR **(a)** and *H19* DMRs **(b).** In the present study, we adopted regions of four DMRs from a previous study by Jang et al. [14]. The imprinted region of *H19* has three DMRs (DMR1, DMR2, and DMR3) and the imprinted region of *Igf2* has one DMR.
