# Supplementary material for: Developmental and Degenerative Characterization of Porcine Parthenogenetic Fetuses during Early Pregnancy
Source: Animals (Basel). 2020 Apr 4;10(4):622. doi: 10.3390/ani10040622 (PMC7222715; doi:10.3390/ani10040622)
Supplement: Supplementary file 1 [file animals-10-00622-s001.zip › animals-742082 -supplementary/Supplementary Table 1-edited.docx]

**Supplementary Table 1.** PCR primer sets for bisulfite sequencing.

| **Regions** | | **Sense primers** | **Antisense primers** |
| --- | --- | --- | --- |
| Igf2-DMR | Outside | GAGGTTTAGGGGTTTAGATTTTTTT | ACCCAACATTTAACAAACCCAACTC |
|  | Inside | GATATTTGGTGGAGTAAGTTTTGTG | CTRTTCCCACCCTRRCTRRAAACAARR |
| H19-DMR1 | Outside | TGGTTAGGGATAGGAGATTAGGTTTA | AATAACAACTACCACTCCCCTCATAC |
|  | Inside | GATATTTGGTGGAGTAAGTTTTGTG | CTRTTCCCACCCTRRCTRRAAACAARR |
| H19-DMR2 | Outside | ATTATTAAGGTTGGGATTTGAGATT | AACCCTATACCACCAAAAACCCTAC |
|  | Inside | AGGTGTTATTTTGTTTGTTGGT | ATAAAATAACCTAAAAAAACTCAA |
| H19-DMR3 | Outside | GGTTTTAGGGGGATATTTTTT | TTAAAAAAACATTACTTCCATATAC |
|  | Inside | GATTTTTAGGTTTGTTATTATTT | CAAATATTCAATAAAAAAACCC |

All primer sets were adopted directly from a previous study by Jang et al. [15].
